# Pott Puffy Tumor in Adults: The Τiming of Surgical Ιntervention

**DOI:** 10.7759/cureus.11781

**Published:** 2020-11-30

**Authors:** Giorgos Sideris, Alexander Delides, Konstantinos Proikas, Nikolaos Papadimitriou

**Affiliations:** 1 2nd ENT Department, Attikon University Hospital, National and Kapodistrian University of Athens, Athens, GRC; 2 2nd ENT Department, School of Medicine, Attikon University Hospital, National and Kapodistrian University of Athens, Athens, GRC

**Keywords:** pott's puffy tumor, sinusitis, osteomyelitis, surgery

## Abstract

Pott’s puffy tumor (PPT) represents a rare complication of frontal sinusitis, and it is considered as a subperiosteal abscess of the frontal bone based on osteomyelitis.

We report two adult PPT patients and discuss the treatment plan as well as the correct timing of surgical intervention. Clinical examination revealed sinusitis with puss, and imaging findings showed bony erosion of the dorsal wall of the frontal sinus in both patients. In case 1, a “wait and see” approach was followed with remission of the patient’s symptoms, and a Draf IIb type was performed 21 days after discharge. In case 2, worsening of symptoms led to surgical drainage through a Lynch incision followed by 20 days of intravenous antibiotic treatment. Then a Draf type IIa was performed. Both patients received antibiotic therapy over the course of six weeks and had full recovery.

We highlight the importance of the correct timing of surgical intervention as it is depended on the clinical and radiological findings. The timing to performing radical drainage surgery including external or endoscopic frontal sinus surgery is not determined in the literature. Worsening of common symptoms and neurological signs in adult PPT patients means by default an immediate surgical intervention. Reduction of symptoms and antibiotic treatment response means that surgery should be delayed and performed in a surgical field free of inflammation.

## Introduction

Two-hundred and fifty-two years after Londoner surgeon Percivall Pott, one of the founders of orthopedics, described a “tumor” believed to be caused by a complication of direct trauma to the forehead, many things have changed. Pott himself reported another similar case seven years later caused by frontal sinusitis. Nowadays Pott’s puffy tumor (PPT) is considered as a subperiosteal abscess of the frontal bone based on osteomyelitis, and it represents a rare complication of frontal sinusitis with or without head trauma [1-4].

PPT is characterized by a circumscribed inflammatory swelling of the forehead presenting with fever, headache, and nasal discharge. Intracranial complications such as subdural empyema, epidural abscess, and cavernous sinus thrombosis may be present [3]. Early diagnosis with clinical examination and imaging is essential, whereas antibiotic treatment and surgical drainage are the standards of care.

We present two cases of adult patients with PPT as a complication of frontal sinusitis and discuss their treatment plans focusing on the timing of surgical intervention.

## Case presentation

Case 1

A 50-year-old male presented at the Otolaryngology emergency department complaining of headache and facial pressure. He reported visiting a provincial hospital 25 days ago with the same symptoms and was treated with oral antibiotics, saline nasal spray, and nasal corticosteroids.

Clinical examination revealed an inflammatory swelling of the forehead that developed the last three days and mild left periorbital swelling. There was no past history of head trauma or other comorbidities. The patient was afebrile, and the lab tests were unremarkable. Nasal endoscopy revealed sinusitis with puss, and nasopharyngeal swab cultures were positive for *Staphylococcus aureus*.

**Figure 1:**
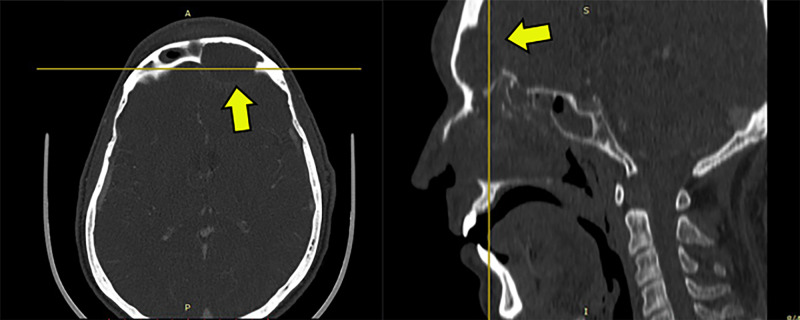
CT scan (Case 1) Contrast-enhanced axial computed tomography (CT) scan showed bony erosion of the internal wall of the frontal sinus as well as the existence of an abscess (yellow arrows).

Contrast-enhanced axial computed tomography (CT) scan showed bony erosion of the dorsal wall of the frontal sinus as well as the existence of an abscess (Figure 1).

**Figure 2:**
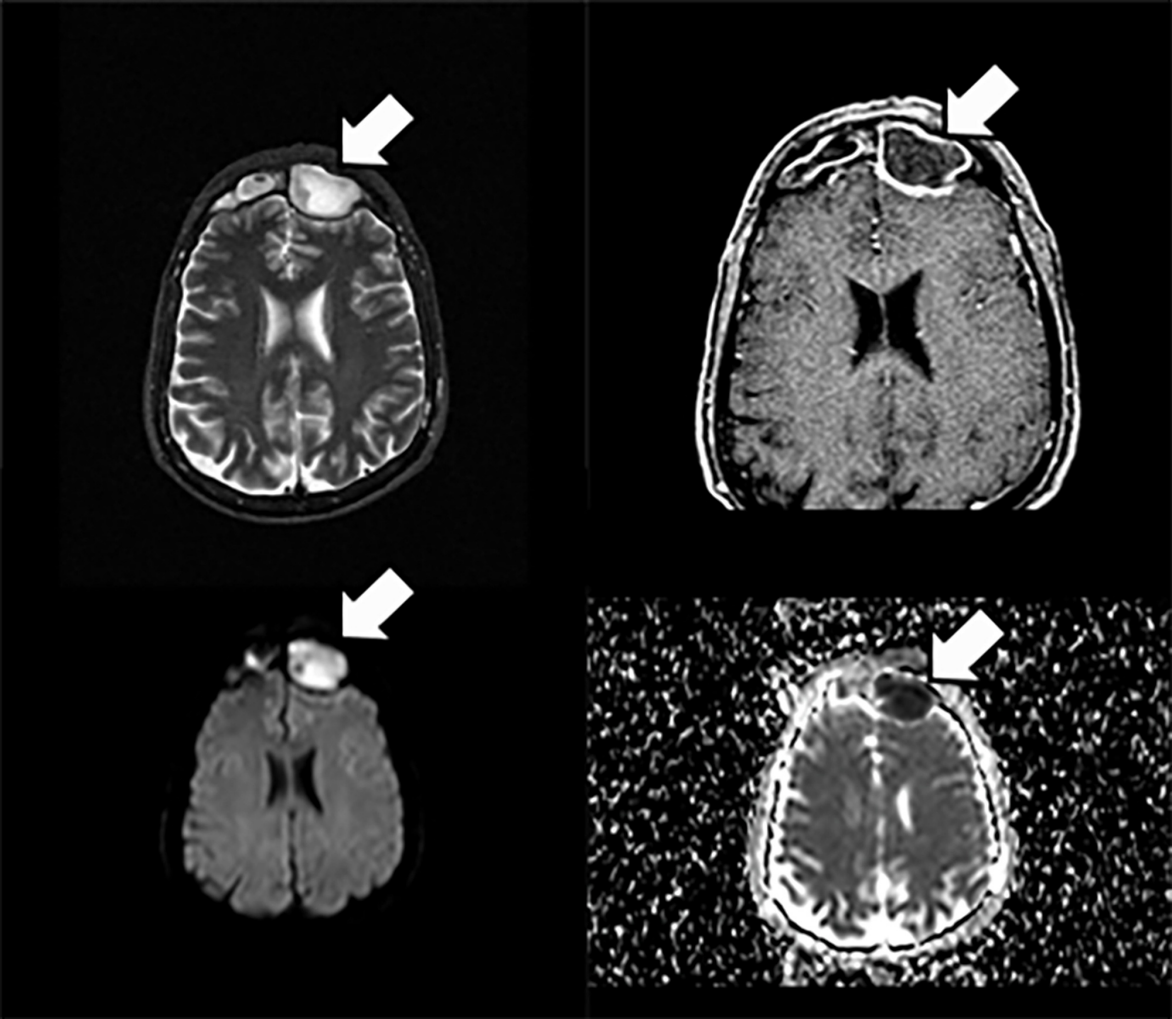
MRI scan (Case 1) Magnetic resonance imaging (MRI) showed enhancing lesion filling the left frontal sinus and erosion of the internal wall of the frontal sinus with extension to the anterior cranial fossa. Short tau inversion recovery (STIR), diffusion-weighted imaging (DWI), and apparent diffusion coefficient (ADC) showing diffusion restriction as well as enhancement and thickening of dura mater (white arrows).

Magnetic resonance imaging (MRI) showed enhancement of the lesion in T2-weighted images, whereas in T1 there was ring enhancement characteristic of a mature abscess (Figure 2). 

Neurosurgical and ophthalmology assessments revealed no neurological or ophthalmic symptoms.

The patient was treated with intravenous (iv) antibiotics for 10 days with remission of his symptoms and reduction of the swelling. Oral antibiotics prescribed on discharge for another 10 days.

After 21 days he was submitted to surgery, and a Draf type IIb was performed. Extended drainage was achieved after ethmoidectomy by resecting the floor of the frontal sinus between the lamina papyracea and the nasal septum.

The postoperative period was uneventful, and the patient was discharged after three days of receiving oral antibiotic therapy for six weeks.

Case 2

Α 47-year-old male with past medical history free of trauma was admitted to our emergency Otolaryngology department complaining of a headache.

Clinical examination revealed forehead swelling, erythematous left eye, and periorbital swelling. There were no neurological or ophthalmological symptoms. Lab tests showed no remarkable inflammatory markers. Rhinorrhea of pus was noticed in posterior rhinoscopy, and a nasopharyngeal swab was collected that was positive for *Staphylococcus hominis*.

**Figure 3:**
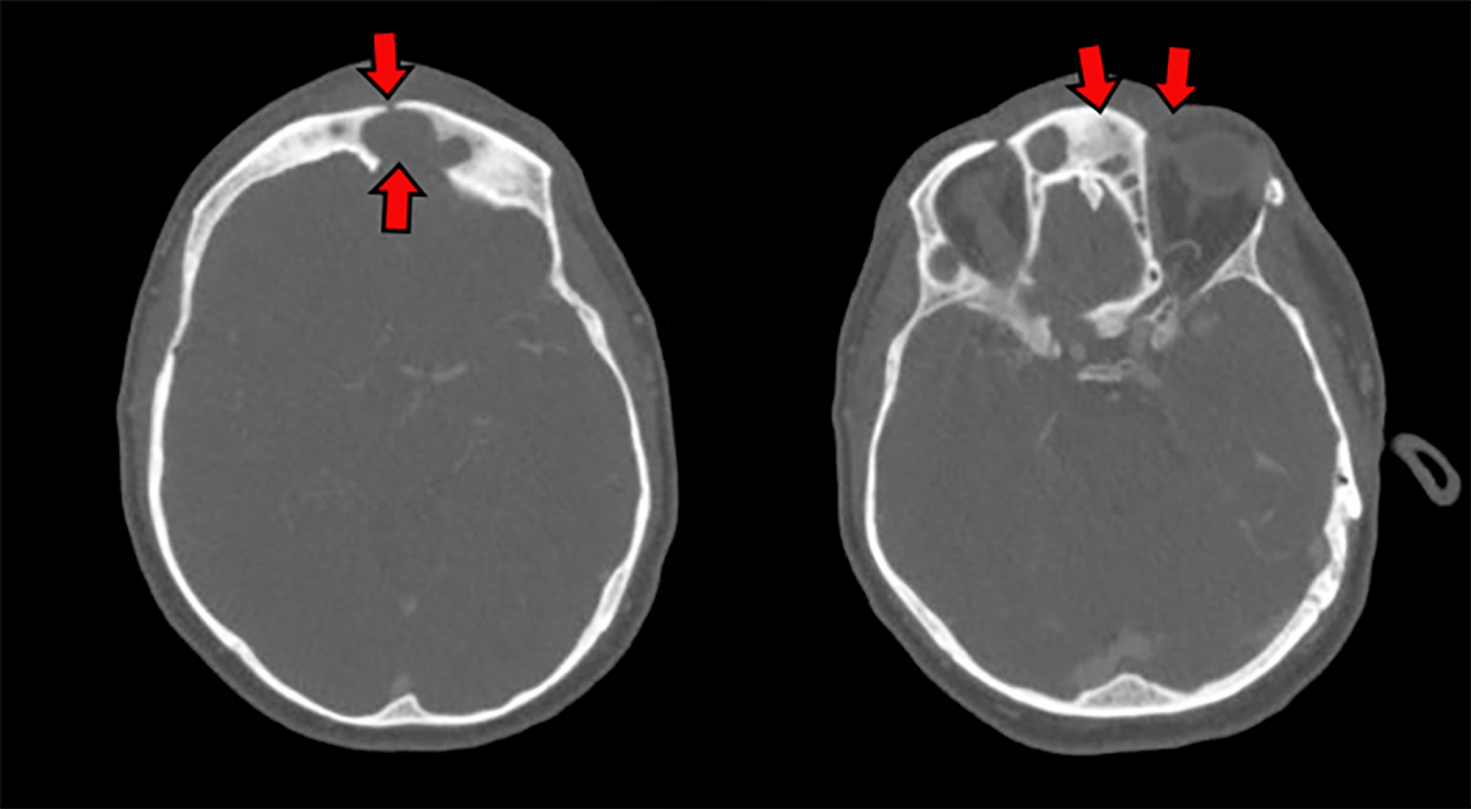
CT scan (Case 2) Contrast-enhanced axial computed tomography (CT) scan showing a large bony erosion of the internal wall of the left frontal sinus and a small bony erosion of the anterior wall (red arrows).

Contrast-enhanced axial CT scan showed a large bony erosion of the dorsal wall of the left frontal sinus and a small bony erosion of the anterior wall (Figure 3).

The patient was treated with iv broad-spectrum antibiotics but had worsening of symptoms with fever and strong headache in the following two days.

**Figure 4:**
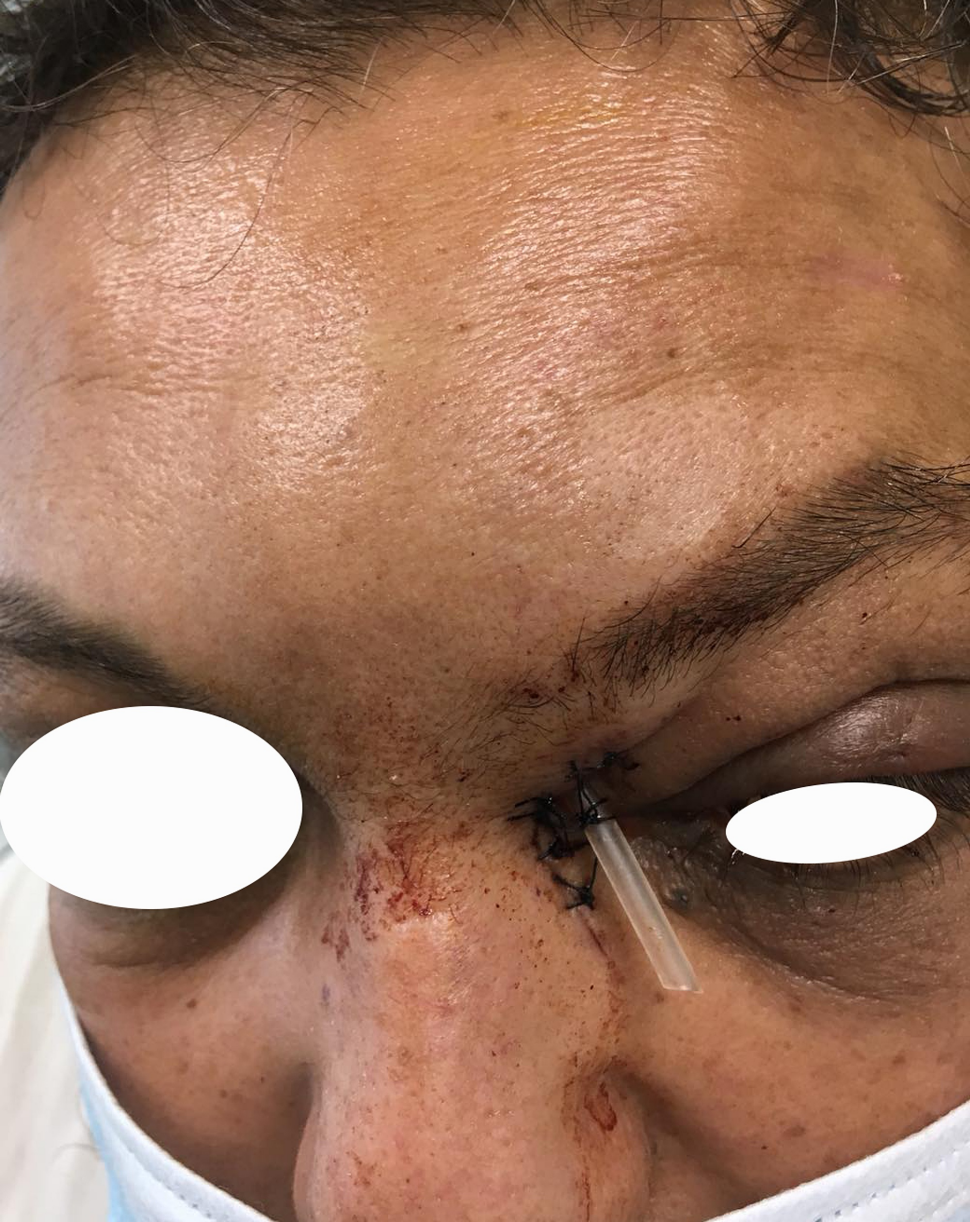
Patient of case 2 Patient 2 on post day 2 after immediate surgical drainage through Lynch incision. Frontal left tumefaction has been increased, and periorbital swelling still exists. A “Penrose type” tube is placed for trauma's daily irrigation.

An endoscopic attempt to drain the abscess through the frontal recess was abandoned due to inflammatory swelling, difficulty in recognition of anatomical landmarks, and intraoperative bleeding. The abscess was drained through a Lynch incision and drill trephination in order to access the medial wall. A “Penrose type” tube was placed, and the trauma was daily irrigated with natural saline and nasal corticosteroids were placed (Figure 4).

The patient continued his treatment with iv antibiotics for six days and continued with oral antibiotics for another 15 days after his discharge. He was taken again to surgery where a Draf type IIa was performed resecting the floor of the frontal sinus between the lamina papyracea and the middle turbinate.

The post-operative days were uneventful, and the patient was discharged on the second post-operative day free of symptoms, receiving oral antibiotic therapy for four weeks.

## Discussion

PPT in adults is a rare and potentially life-threatening complication of frontal sinusitis that relates to delayed or inadequate treatment. It is not the aim of this paper to focus on the clinical, imaging, and laboratory findings of adult patients with PPT. After all, our patients' clinical findings are similar to the international literature with headache being the most common symptom [3]. None of the patients were febrile, and PPT presented as a forehead inflammatory swelling following frontal sinusitis without a history of head trauma.

Our imaging findings revealed that both of the patients had a mild intracranial extension with no neurological symptoms and without subdural empyema, brain abscess, cortical vein thrombosis, and epidural abscess although some studies report that intracranial complications appear in 29% of the adult patients with PPT [5]. Laboratory tests had no significant results. We confirmed in both cases that the most common pathogen microorganisms isolated are *Staphylococcus *species [6]. Differential diagnosis of PPT includes soft-tissue infections as well as benign and malignant tumors [7,8]. Final diagnosis can be established by clinical and radiological findings. CT scan is useful in revealing the presence of epidural or subdural abscess and to estimate the extent of bone erosion, whereas MRI scan can detect intracranial involvement [6,9].

There is a variety of treatment approaches in the literature, but guidelines on treatment plans and the correct timing of intervention have not been discussed enough.

Ketenci et al. presented six PPT patients and suggested that supraorbital rim or glabellar incision is enough for the drainage of abscesses, followed afterward by endoscopic ethmoidectomy with frontal sinusotomy in order to remove ostiomeatal complex pathology [6].

Sekine et al. reported that surgical treatment depends on the severity of the infection, with intracranial extension alerting for emergency craniotomy and frontal sinus cranialization after total removal of the sequestrum. The same authors suggest that in the absence of abscess or intracranial and orbital complications, treatment should be based only on antibiotics [10].

In a series of six patients, Van der Poel et al. reported that endoscopic frontal sinusotomy (Draf IIa) is a viable and safe approach for the surgical management of PPT. The authors suggested that treatment can be delivered endoscopically and, if there are intracranial complications, with a burr hole [11].

In a review of 32 patients, Akiyama et al. reported that external surgical procedure was chosen in 58.1% of cases, but endoscopic sinus surgery was frequently selected in 32.9% of reports, although external subperiosteal abscess drainage was needed in all of those cases. Only forehead drainage treatment was performed in three cases without radical drainage surgery for frontal sinuses. Authors also report that the optimum period for performing radical drainage surgery, including external or endoscopic frontal sinus surgery, has not been determined [5].

The timing and strategy of treatment raise some questions. Is there any correlation between the time from symptoms and swelling onset to surgery? Is it more prudent to “wait and see”? After all, PPT is a complication of frontal sinusitis, and we must treat it as bacterial sinusitis.

Akiyama et al. reported that the average time for radical operations was 6.8 weeks, and no significant difference was observed between positive and negative groups for intracranial complications [5].

Our experience confirms the literature recommendation that combined treatment with surgical intervention and broad-spectrum antibiotics has the favorable outcome [12]. Existence of intracranial complications, neurological clinical signs, and worsening of common symptoms such as headache 48 hours after iv antibiotic therapy means by default urgent surgical drainage (with external or endoscopy approach) as in case two. According to the literature, intracranial extension is not always requiring neurosurgery intervention [13]. Based on our clinical experience in PPT cases with gradual resolution of symptoms and no intracranial complications treatment with iv broad-spectrum, antibiotics should be the first choice, and the surgical intervention should be scheduled after a minimum of 20 days so that the inflammation levels have been reduced, as in case one. We confirm literature recommendations for antibiotic therapy over the course of six weeks [14].

## Conclusions

PPT is a complicated frontal sinusitis, osteomyelitis of the frontal bone, and represents a rare entity in adults with severe intracranial complications. Swelling of the forehead and headache should represent a PPT; therefore, one should keep a high suspicious index. Diagnosis is based on clinical and radiological findings. Multidisciplinary management with neurosurgical and ophthalmology assessment is always recommended. Worsening of common symptoms and neurological signs represent a red flag emergency that needs immediate surgical intervention. Surgical approaches are depending on the extension of the lesion (external, endoscopy, and neurosurgery). Reduction of symptoms and antibiotic treatment response means that surgery should be delayed and performed in a surgical field free of inflammation.
